# Nationwide Survey on Neonatal Critical Congenital Cardiopathies in Mexico: Data from 76 Public Health Service Hospital Units

**DOI:** 10.3390/ijns11020046

**Published:** 2025-06-16

**Authors:** Nina Mendez-Dominguez, Ely Sanchez-Felix, Joan Johnson-Herrera, Miguel Santaularia-Tomas, Andres Ku-Gonzalez, Luis Baeza-Herrera, Adriel Ismael Alonso-Batun, Marcos Rivero-Peraza, Humberto Camara-Conde, Amonario Olivera-Mar, Russel Camara-Beltran

**Affiliations:** 1 IMSS-Bienestar, Hospital Regional de Alta Especialidad de la Peninsula de Yucatan, Mérida 97130, Mexico; esanchez.hraepy@imssbienestar.gob.mx (E.S.-F.); joan.jhonson@imssbienestar.gob.mx (J.J.-H.); msantaularia@imssbienestar.gob.mx (M.S.-T.); andres.ku.gonzalez@gmail.com (A.K.-G.); luisbaezaherrera@gmail.com (L.B.-H.); aiab.231@gmail.com (A.I.A.-B.); amar.hraepy@imssbienestar.gob.mx (A.O.-M.); 2 School of Medicine, Universidad Anahuac Mayab, Mérida 97302, Mexico; 00380342@anahuacmayab.edu.mx; 3 School of Medicine, Universidad Marista de Merida, Mérida 97300, Mexico; hcamara2014052@a.marista.edu.mx; 4 School of Medicine, Universidad Autonoma de Yucatan, Mérida 97000, Mexico; psic.russellcamara@gmail.com

**Keywords:** newborn, cross-sectional studies, Mexico, heart defects, congenital, hospital units

## Abstract

When the resources are available, critical congenital heart diseases (CCHDs) should ideally be detected in utero; however, their later detection at birth can still reduce negative outcomes and risks. This study aimed to assess the extent of cardiac screening implementation in a national sample of hospitals within Mexico’s public health services. A cross-sectional survey was conducted to identify the barriers and facilitators to neonatal screening using a sample of 76 hospitals. The descriptive statistics and associations were analyzed, with significance set at *p* < 0.05. Only 12% of hospitals reported the routine implementation of CCHD screening, while 20% used variable screening criteria. A potential mandatory implementation of CCHD screening was associated with increased odds of perceiving the lack of protocols and guidelines as a barrier. The most frequently reported obstacles involved a lack of the following: equipment, designated physical space, trained personnel, and adequate training. Nevertheless, the facilitators identified suggest that when combined with standardized guidelines and protocols, routine nationwide implementation may be achievable.

## 1. Introduction

The right to health must be accessible to all humankind from the beginning of life; for this right to be ensured, there must be congruent ways to deliver it [1].

Although in Mexico, as in the rest of the world, there are pathologies whose prevention and timely detection are considered imperative due to their high morbidity (e.g., diabetes), there are also infrequent conditions that due to their severity, progression, or lethality require the same attention to be prevented and detected in a timely manner, such as in the case of critical congenital heart diseases (CCHDs) [2]. When the means are accessible, these should be detected in utero; if this is not feasible, congenital cardiopathies must be detected at birth, which allows patients to be offered the optimal resources to address them promptly and accurately. The later the suspicion of congenital heart disease becomes apparent in an affected child, the greater the probability of negative outcomes. In turn, this means that invasive interventions have a lower probability of success and a greater risk of disability and lethality [3,4].

More than one individual life is affected by the late diagnosis of a CCHD; it means a painful process not only for the affected child but also for each member of the family [5]. For all these reasons, the routine screening of newborns for congenital heart disease is justified in terms of their right to health, as well as for the optimization of resources and the limitation of damage.

In 2011, following a statement by the American Academy of Pediatrics (AAP) and the American Heart Association (AHA), a multidisciplinary panel of experts was convened to discuss strategies for integrating pulse oximetry screening into hospital neonatal practice protocols—a method that, in the same year, was added to the Basic Uniform Screening Panel. This recommendation has had a significant impact on various countries [6,7,8].

Neonatal pulse oximetry screening measures the level of oxygen saturation in neonatal blood during the first hours of life; this can be carried out by any member of the hospital staff that is trained in this procedure in order to detect any degree of hypoxia that may have been missed during physical examination due to a lack of visible manifestations of hypoxia [9,10]. To perform this test, a pulse oximeter that has previously been calibrated for use in neonates and low perfusion conditions and that has a root mean square error accuracy of 2% is used to detect functional oxygen saturation. This test should ideally be performed during the first 24 h of life; however, if this is not possible, it is essential to perform it before the patient is discharged. The oxygen saturation level should be taken in the upper limbs (right hand) to obtain pre-ductal saturation, as well as in the lower limbs (either foot) for post-ductal saturation. If the test results in a saturation of ≥95% in both limbs, as well as a difference of ≤2% between pre- and post-ductal circulation, the test is considered negative. In the case of a saturation of 90–94% or a difference of ≥2% between pre- and post-ductal circulation, the test should be repeated within an hour after the first take. If, after repeating the test, the values continue to be within the same range, the test will be considered positive. Nevertheless, the test is considered positive if saturation is <90% during the first pre- or post-ductal circulation assessment [11].

Pulse oximetry is cost-effective, non-invasive, safe, and easy to perform since it has been reported that the examination and follow-up have an approximate cost of USD 1–10 per neonate, a duration of <1 to 5 min, and can be performed by any member of the hospital staff. The performance of pulse oximetry in neonates, depending on the study, has presented a sensitivity of between 62 and 76%, as well as a specificity >99%, for the detection of CCHDs; additionally, when used in conjunction with auscultation, a sensitivity of 92.3% has been reported [12]. On 28 April 2021, in Mexico, Congress approved a national decree indicating that screening for critical or severe congenital cardiopathies before hospital discharge for all newborns is compulsory [13]. In November 2022, the public health Institution IMSS (Mexican Institute for Social Insurance in Spanish) released some technical guidelines relating to neonatal cardiac screening in the mentioned institution units [14].

There are known limitations to the universal use of CCHD screening devices across regions that go far beyond norms and device costs. First, in developing countries, pregnancy control may not start as often in the first trimester, and women may still give birth at home, aided by female family members; therefore, their offspring may not have access to screening until taken for neonatal assessment. However, neonatal external consultation can still mean an opportunity for screening [15].

Through the timely identification of CCHDs in children, cardiac conditions may be properly addressed and other extracardiac conditions can be identified/monitored; for example, in Colombia, a medical/surgeon team evaluated 378 neonates with critical congenital heart diseases, finding that 69.3% also suffered from extracardiac conditions [16].

The objective of the present study is to establish the extent of the implementation of screening for critical congenital heart diseases in a nationally representative sample of medical units from the public health services in Mexico.

## 2. Materials and Methods

This study presents a cross-sectional survey that was conducted between October and December 2024. The questionnaire that was used was adapted from the one conducted by Brown, Liyanage, Mikrou, Singh, and Ewer in the United Kingdom’s neonatal units by N.M.-D., M.S.-T., E. S.-F., and J.J.-H. and validated by A.O.-M., R.C.-B., and A.K.-M. We included information about the location and characteristics of the medical units [17]. The study’s total population was based on the reported infrastructure of the public health service institutions, comprising 82 second- and third-level hospitals [18]. Our sample was composed of those hospitals that attended births or offered neonatal care (N = 76). The hospitals were included if they participated in the provision of public health services but were excluded if they were in the process of changing the medical services that they offer (after first assessment by H.C.-C., M.R.-P., and A.I.B.-A.).

To ensure single responses per unit, as well as the accuracy of the individuals providing responses, H.C.-C. and M.R.-P. generated an online form that was restricted to users of IMSS-BIENESTAR mail and delivered by N.M.-D. using Microsoft^®^ Forms and reminders were performed by phone calls to each hospital/unit. The survey was derived from a project that was approved by institutional ethics and research review boards (2024-016) for the distribution of the questionnaire; permission was obtained from the Medical Research Division.

The items in the questionnaire were closed-ended, and were later transformed into either dichotomous, categorical, or ordinal variables; additionally, an optional comments section was enabled. We asked about the resources in the hospital and the related hospital services (e.g., obstetrics and neonatal consultation), as well as whether CCHD screening was implemented and, if so, how regularly. We explored the hospital services where screening was implemented (and due to be implemented), as well as asking about the barriers and facilitators for achieving screening implementation in the hospitals/unit.

Once seventy-six responses were collected (on 13 December 2024), the online form was locked, and no new responses were received. The responses were imported to a spreadsheet before being codified and further processed by R.C.-B. and H.C.-C. For the descriptive statistics, we obtained frequencies and percentages; the numerical data are presented as a discrete sum. We graphed the location of each respondent unit, grouped according to state on the map. We performed binary logistic regressions with post hoc goodness-of-fit tests for analyzing the characteristics of the units that had implemented screening and those that had not. We also analyzed the odds for identifying the facilitators, given the nature of the perceived barriers, as well as in the case of CCHD screening becoming mandatory. For all cases, confidence intervals were established at 95% and significance was established at *p* < 0.05 using Stata 14; data processing and statistics were performed under the supervision of N.M.-D. and M.S.-T.

## 3. Results

**Figure 1:**
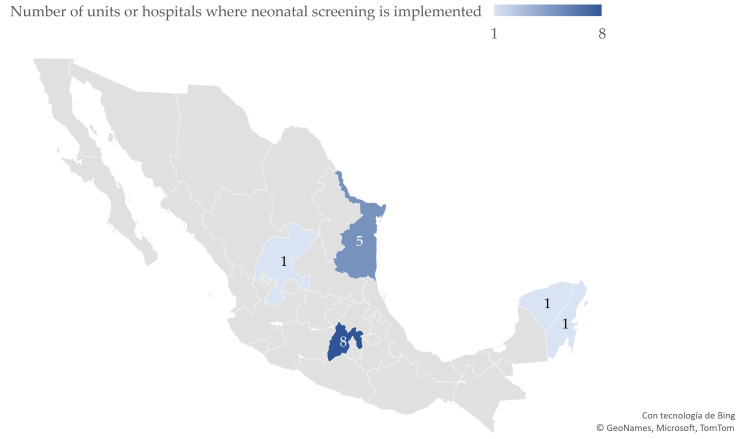
Distribution of public hospitals/units where screening for critical congenital heart diseases has been implemented in Mexico (N = 76 hospitals/units).

Of the 76 respondent hospitals/units, 66 assisted in childbirth and 10 provided medical services where CCHD screening could be implemented; of the 16 units (20%) referring an implementation of neonatal cardiac assessment, 7 (9.2%) had variable screening criteria and 9 (12%) implemented routine CCHD screening for all neonates. The geographical distribution of these hospitals/units is shown in Figure 1. Of the units that could perform CCHD screening, 14 reported screening could be implemented in the neonatal–maternal side room, while 10 mentioned external consultation relating to neonatal or preventive services. Of the 52 remaining units, only 16 were identified as unit departments where screening could be performed, while 4 units reported a lack of a specific physical area to perform the screening. The distribution of units implementing CCHD screening is shown in Figure 1.

Neonatal oximeters are available in twenty units (26.32% ± 4.3); however, only one unit (1.64% ± 1.2) has plans to implement screening in the following six months. In total, there are 37 units that do not perform CCHD screening; these units mentioned that they cannot implement screening because they do not have the devices. Furthermore, 29 units reported having no trained personnel, 7 units perceived the lack of norms or guides as a barrier, 11% mentioned not knowing how to refer patients testing positive as a barrier, and another 4 units perceived the probability of false positives as a barrier, as shown in Table 1.

The most frequently identified facilitator was the provision of oximeters (*n* = 59), followed by personnel training (*n* = 56); in total, 43 units were considered to have higher levels of personnel and normative protocols as potential facilitators.

A total of 32 units had a specific physical area to perform screening, while 21 units considered that having more reliable device options could facilitate the implementation. A median of three main barriers and four possible facilitators was observed from all respondent units. In general, 13 respondents consider it highly feasible to implement routine CCHD screening in the short term, 24 in the medium term, and 39 in the long term. Nevertheless, if the perceived facilitators are not achieved, 13% of units perceive a low probability of implementation, even for the long term, as presented in Table 2.

As concerns the analysis of responses in the survey, comparisons between units that routinely perform CCHD screening were not well adjusted in post-estimation tests, indicating an unbalanced frequency for estimating association measures through logistic regression.

The perceived probability for implementation in the long term was analyzed in association with the sum of the perceived barriers and the assumption of achieving potential facilitators, as shown in Table 3.

Additionally, the association relating to a potential mandatory implementation of CCHD screening in the participating units would increase the odds of perceiving the lack of protocols and guides—along with the false positives being considered to be significantly problematic—as shown in Table 4.

Finally, additional comments led us to understand why some units that have already implemented CCHD screening still perceive barriers to routine screening. In two units, the neonatal oximeters are damaged or lack certain pieces/attachments; others have used the units, which were only placed in the hospital temporarily as part of a joint project with local universities. In one hospital, the nursery was not considered ideal for screening because neonatal nursing staff would not compromise to perform more duties than they already do. In addition, comments from the survey included the fact that the devices are not accurate and that the units would not like to communicate false and alarming information to the newborn’s parents.

## 4. Discussion

The present study highlights a problem for delivering a prompt diagnosis in children with neonatal congenital cardiopathies, as perceived in a national sample of hospitals/units from the public health services of IMSS-BIENESTAR.

Regarding the extent of the routine implementation of CCHD screening in the units studied, we found that even when 20 units have equipment for testing oximetry in neonates, only 15 of them have been implementing the screening on a routine basis. This may be due to other aspects that are unrelated to device availability, such as protocols, designated personnel, and damaged equipment. Even when respondents specified that having devices for oximetry is the principal potential facilitator, it would need more than just the equipment for a routine screening to be implemented in all units. The low prevalence of CCHDs means that more complex and organizational efforts are needed; large implementation studies are required to show statistically significant improvements in newborn outcomes, such as those reported since the implementation of screening in the US [19]. Therefore, the acquisition of devices is possibly only one step that leads to significantly improving the registries and prognoses of children with CCHDs.

Training for these roles and responsibilities, along with a stipulated designated area, would help organize hospital teams to work together and implement routine CCHD screening in the unit. However, these roles and responsibilities may benefit from general norms, protocols, and guides that could be discussed and established in an expert working group with the participation of decision-makers, program designers, and implementation personnel. These aspects have also been previously described as opportunities for improving neonatal CCHD screening [20]. Some responders considered the need for additional personnel to perform cardiac screening as a limitation. Previous studies show that rather than including additional personnel for screening, the existing nurses can be trained in situ for implementing screening into their existing workflow [21]; in specific cases, where doubts arise, telemedicine can also be used [22].

Hospitals/units contemplating the implementation of screening were also expected to release protocols and guides, which is consistent with the findings of the UK survey where 66% of units were willing to implement screening but were waiting for national recommendations relating to such a process. Staff allocation and training personnel for screening were also aspects mentioned in the UK survey that were reported by our respondents. However, the physical area in which to perform the screening was not a concern that was raised in the mentioned survey [17].

We believe that false positives may be considered problematic because the personnel are not sure how to handle and refer the positive patients for more specific studies such as echocardiography. Additionally, it may require the units with neonatal echocardiography facilities to perform the study before discharge, as well as having to train pediatricians in relation to soft skills in order to correctly provide accurate information to parents without generating an unnecessary alarming diagnosis. Training in the correct screening procedure, combined with auscultation and imaging studies, is as important as training for clear communication with parents; therefore, both training strategies are needed [22].

The underdiagnosis of congenital heart disease still exists in Mexico; this is supported by a recent review by Van der Linde et al., which states that health care and early diagnosis are still unequally distributed worldwide and translate to the differences in CCHD rates reported at birth between high- and low-income countries—even when genetic, environmental, socioeconomical, and ethnic determinants may play a role [23]. By implementing mandatory CCHD screening at hospitals/units in Mexico, we may contribute to reducing the gap, estimating incidences and prevalences as accurately as possible, and allocating proportional resources to address this uncommon but relevant health problem.

Finally, mandatory CCHD screening cannot translate into a routine practice in hospitals/units unless it is functional and complete, as well as adequate equipment being allocated in every unit receiving births or providing neonatal consultation. This can only be achieved through funding either for the design of new devices or for the acquisition of existing models on the market. A study exploring the strategies employed in other countries when their implementation began [24,25] has the potential to orient multidisciplinary experts, stakeholders, and decision-making panels to define and decide which of them could be congruent with the public health services in Mexico. Moreover, quality assessment and evaluation plans will be needed for continuous improvement.

## 5. Conclusions

Certain hospitals/units in Mexico have implemented routine critical congenital cardiopathy screening for neonates in the public health services of the studied hospitals. Roughly 12% of the units perform screening on a regular basis in neonates. A lack of equipment, physical area, personnel, and training have been identified as barriers for screening implementation; however, the potential facilitators described could jointly derive into routine implementation nationwide if combined with mandatory indications and complementary protocols, guides, and referral indications that are congruent with training for health personnel, including second-level hospital medical doctors. To reduce the perceived barriers relating to screening implementation, the acquisition of oximetry devices for hospitals/units should be addressed, along with the development of more precise oximeters, which could reduce concerns regarding false-positive results.

From our results from the public hospitals/units, we conclude that the implementation of neonatal screening for congenital cardiopathies is not equally distributed across Mexico. Efforts are needed to achieve democratization of neonatal screening, with equal opportunities for a timely diagnosis for both private and public health institutions, to benefit all neonates born in Mexico regardless of their place of birth or economic status.

## Figures and Tables

**Table 1:** Perceived barriers and potential facilitators as reported by responders of 76 hospitals/units from Mexican public health services.

Responses	Number of Hospitals/Units	Percentage
Barriers		
Lack of equipment	59	77.63
Do not have enough personnel	48	63.16
Do not have qualified personnel	35	46.07
Inability to identify where or how to refer	11	14.47
No normative protocols	9	11.48
False positives may be problematic	4	5.26
Facilitators		
Having a neonatal oximeter	59	77.63
Trained personnel	56	73.68
Having more personnel	43	56.58
Establishing protocols and guides	43	56.58
Having a specific physical area	32	42.11
More reliable neonatal oximeters	21	27.63

**Table 2:** Characteristics of the 76 hospitals/units according to the implementation of critical congenital heart disease screening.

Characteristics of the Hospitals/Units (N = 76)	Number of Hospitals/Units	Percentage
Have no plans for implementation in the following six months	36	47.54
Have devices for oximetry	20	26.32
Plan to implement within the following six months	10	1.64
Do not perform CCHD screening	52	68.42
Perform CCHD screening on some occasions	15	19.74
Have implemented routine CCHD screening	9	11.84
Have established a departmental area for screening	24	31.58
Screening is performed in a maternal–neonatal side room	14	18.42
Have an obstetric nursing unit	66	60.66

**Table 3:** Binary logistic regression relating to the perceived probability of implementing critical congenital heart disease screening in the long term.

Probability of Implementation	Odds Ratio	Standard Error	Z	*p*	95% Confidence Interval	
Barrier sum	0.30	0.12	−3.05	0.00	0.14	0.65
Facilitators achieved	1.67	0.36	2.37	0.02	1.09	2.55
Mandatory implementation	14.95	17.63	2.29	0.02	1.48	78.76

Pseudo R^2^ = 0.15; post hoc Hosmer–Lemenshow = 0.32.

**Table 4:** Binary logistic regression relating to the odds for perceived barriers if mandatory implementation was indicated according to the responses from 76 hospitals/units regarding the implementation of critical congenital heart disease screening.

Barriers that Responders Believe Would Arise from Mandatory Implementation	Odds Ratio	Standard Error	Z	*p*	95% Confidence Interval	
Barrier of lack of protocols and guides	24.00	29.13	2.62	0.009	2.22	25.91
Barrier that false positives may be problematic	29.82	46.37	2.18	0.03	1.41	62.84

Pseudo R^2^ = 0.12; post hoc Hosmer–Lemenshow = 0.84.

## Data Availability

Data is available upon request to first author.

## Abbreviations

The following abbreviations are used in this manuscript:

CCHD	Critical congenital heart disease
AAP	American Academy of Pediatrics
AHA	American Heart Association
IMSS	Mexican Institute for Social Insurance (Instituto Mexicano del Seguro Social)
IMSS-BIENESTAR	A public health program under the IMSS
